# One Health Requires a Theory of Agency

**DOI:** 10.1017/S0963180122000044

**Published:** 2022-10

**Authors:** Benjamin Capps

**Affiliations:** Department of Bioethics, Faculty of Medicine, Dalhousie University, Halifax, Nova Scotia B3H 4R2, Canada

**Keywords:** one health, public health, agency, anthropocentric, neuroethics, environmentalism, rights

## Abstract

One health suggests that human and animal health are comparable, but in practice, the concept aligns with the principles of public health ethics. One health ethics, as such, appears to eschew connotations of equality for the natural world. A theory of agency revises that anthropocentric assumption. This article begins with a critique of environmental dualism: the idea that human culture and nature are separate social realms, thus justifying public health as a (unifying) purpose. In response, this article argues that, first, a neuroethics of one health might equally regard humans and (some) animals, which have comparable mental states, as rational agents. Second, rational agency should ground our moral connections to nature in terms of the egalitarian interests we have (as coinhabitants) in the health of the planet. While this article makes a moderate case for interspecific rights (as the first argument asserts), neuroscience is unlikely for now to change how most public institutions regard nonhuman animals in practice. However, the second argument asserts that rational agency is also grounds for philosophical environmentalism. One health ethics, therefore, is a theory of equality and connects culture to nature, and, as such, is a separate, but coextensive approach to that of public health.

## Introduction

The contemporary one health movement has been defined as an instrumental approach to augmenting public health by enhancing health at the human–animal–environment interface.^1^ Variations of this anthropocentric view are now common in national and global responses claiming to use the one health approach.^2^ On the one hand, if one health *is* merely descriptive of disciplinarity in contexts where humans interact with their environments, then further normative discussion is perhaps redundant.^3^ In this respect, the collaborative approach to one health has been cautiously welcomed, but critically relies on the assumption that “optimal health” is exclusively a matter for human beings, despite other plausible interests at the environmental nexus.^4^ On the other hand, one health ethics also alludes to interconnections or interdependencies between species, and shared interests in the places they live.^5^

In this article, one health ethics is framed as a philosophical critique of environmental dualism. Dualism is the claim that “human culture” and “nature” are separate legal, moral, and social realms. In this respect, I presume that ethical biases toward cultural (or social) determinants of health influence one health as it is currently practiced, thus defining its scope as an environmental philosophy. Although there is perhaps some agreement that the intrinsic value of animals and the environment must be considered in one health, there is uncertainty about their value as compared to the value of humanity. Such issues are, therefore, (unconsciously or conveniently) positioned within public health ethics. So, while a pragmatic case for one health infers that human culture remains a disassociated phenomenon from nature^6^ (and speaks to enhancing public health), a critical view suggests that the environmental challenges we now face are a consequence of anthropocentric models that are indifferent to our *moral* presence in the natural world.^7^

In the first section, I sketch one sense of environmental dualism as rooted in questions about “having moral status.” In this respect, although different species have distinct capabilities and socialities, there are also ways to conceptualize similar states of health, and to conceive of their shared interest through habitat loss, globalization, land use, and climate change.^8^ These connections raise neuroethical implications about agency: specifically, *rational nature* objectively conceives of conditions for moral rights claims, and so defines our duties to modify our behavior such as to try to ameliorate unjust environmental consequences. Thus, in the second section, I argue that if there are plausible analogous mental states between species, then there are also comparable interspecific interests in the conditions for health, so that in theory we ought to equally regard mutual benefits for humans *and* such animals.^9^ However, a neuroscientific basis for one health is likely to be constrained by impracticability, which will become evident if it in theory disrupts practice rooted in the anthropocentric outcomes of public health. So, in the third section, it is suggested that agency (as a neuroscientific phenomenon) also becomes a “fulcrum against which the world might be moved”^10^: *reasonable* agents, as such, have a moral point of view orientated toward “nature.” Nature, therefore, is *necessarily* a vital place for both persons and for other coinhabitants of Planet Earth. This article suggests that agency underlies one health ethics as a rational approach to our relationship to nature.

## Culture and Nature

The disconnect, separateness, and otherness between “culture” and “nature” is a long-standing debate (across multiple disciplines).^11^ Such duality has often underpinned societies’ treatment of animals and the environment, through dichotomous boundaries of “soul” and “soulless,” “cognition” and “automata,” “mind” and “sentience,” and “rational” and “animalistic.” For my purposes, culture may be defined as *inter alia* creation, storage, and implementation of socially learned information, outcomes of high levels of cause-and-effect reasoning, and a capacity to undertake sophisticated cooperative action.^12^ That begs the question: What is it about *human* culture that makes it sufficiently morally different from the animalistic lives around us? And if a difference exists, how should we regard nonhuman nature as separate from our own human kind?

Dualists might agree that human culture is a post-evolution progression when measured in terms of magnitude and hyperdevelopment.^13^ Our ultrasociality is evident in our complex institutions, which require complex laws and moralities to govern.^14^ These accomplishments arise only in the brains of human beings; neurology, therefore, must give rise to the perfection of humanity and the imperfection (or brute physical force) of nature.^15^ This uniquely human characteristic suggests that cultural artifices, such as public health, are only possible because of presence of humanity’s self-creation in the transformation of our environments. In contrast, nature, under this narrative, appears to be spontaneous rather than rational, unaffected by the presence of mind, and wild and uncultivated (I would also note that traditionally “nature” is illustrative of an impossibly contested notion, and there are few concepts as slippery). The point of this particular narrative, however, is that nature—the natural world and nonhuman nature—is instrumental to human well-being; it is meant to be inhabited, used, and manipulated.^16^

Environmental dualism is a socio-legal phenomenon, illustrated, for example, by the ongoing debate about purposively recognizing animal rights by extending “legal personhood” beyond human beings.^17^ In this respect, it might be said that a legal system conceived by and for human interests, is reasonably unrelatable to animals’ interests or the telos of the environment.^18^ Similarly, the science of public health is increasingly understood in terms of the social determinants of health; as such, public health creates a specific perspective about tackling health inequalities, as they follow an experiential gradient with respect to the conditions under which persons are born, grow, live, work, and age.^19^ Health, in this sense, is a cultural phenomenon that includes *environmental* barriers to persons experiencing control in terms of successful agency. Dualists think that animals do not have these experiences (and therefore have no need for public health, social services, or economic and legal insitutions), and, as such, human interests are “alienated” from nature.^20^ Although this infers speciesism, the culture–nature gap may be bridged through modified environmentally friendly institutions, where nature is a passive beneficiary of such ethical reconnection.^21^

Public health may be defined as a superstructure built on *human* rights, in terms of its focus on individual responsibility and collectivism and collective rights generated by the public good.^22^ The principle of *the public interest*, therefore, describes the processes that secure such goods beneficial to communities, humanity, and the human species.^23^ Nature is therefore largely an ecosystem service; and natural elements, such as companion animals, working animals, and natural esthetics (the awesome beauty of nature), are defined through a lens of human well-being.^24^ In these respects, a notion of *cultural environmentalism* ^25^ is the primary anchor for relevant aspects of public health ethics, which corresponds to a focus on controlling our environments, mitigating degradation of spaces, and avoiding resource depletion that eventually affects all of humanity. Ethical inferences in this regard are made from anthropocentric doctrines about population health and preventative medicine.^26^ Environmental solutions are found in the conditions for economic growth (i.e., greening economies and sustainable ecoservices) and sociality (e.g., understanding social connections to nature).^27^ As a result, a broad range of environmental perspectives emerge from social justice lenses,^28^ so that environmentalism carries assumptions about “who benefits” (e.g., from a green economy) and “who bears the burdens” (e.g., of pollution).^29^

Dualism, therefore, defines the “boundaries of the moral community [that] are coextensive with the boundaries of the perceived social community. And sentient beings, so far as [Charles] Darwin knew, did not form a community with man [sic].”^30^ Except animals are part of our contemporary communities—as socially constructed companions, workers, livestock, and pests.^31^ Framing one health as an anthropocentric narrative creates inconsistencies; for example, humans as persons may be “vaccinated” to a disease (and we debate the autonomy of choice and the public interest), but in the same instance, animals as nonpersons are “culled” to protect public health or economies. These potentially superfluous conditions may be described in normal language as discriminatory toward some species or (nonhuman) individuals.^32^ Moreover, culture—human activity—is at the root of excessive and exploitative demands on ecoservices; it justifies lowering animal welfare for economic reasons, promulgating psychological and physiological cruelty; and disregards the bonds between humans and nonhuman companions.^33^ Culture creates animal markets and trade, factory farms, and the entertainment industry, which are potentially causal to our present environmental crises.

*Philosophical environmentalism,* therefore, may be used to critique the adaption of public health frameworks that normally eschew substantive environmental theories; principally, it is a method to cast doubt on presumptive dualism and the assumption that there can be no consilience between theories of human health and animal health. In this respect, we can define the *environmental* determinants of health, such as exposure to pollution and chemicals (e.g., air, water, soil, and products), physical exposures (e.g., noise and traffic), the built environment, and other anthropogenic changes (e.g., climate change), as affecting “cultural” and “natural” beings alike. In this regard, a philosophy of one health may relate to species—connected as if having “one” interest, in the sense of inhabiting the same places and experiencing similar conditions of “health.” Therefore, if dualism *presupposes* that in this shared planetary space there are ostensibly only moral claims to promote human interests, then one health might diverge greatly from the anthropocentric vision of public health.^34^

## The Neuroethics of One Health: An Argument for Interspecific Rights

One area where dualism persists is in neuroethics. For example, a dualist might argue that cognitive mental states (of the kind “I” know exist in “my” mind) are not found in “unconscious” nature; culture, in this respect, requires a noosphere or “thinking arena,” and that creates our unique place outside of the natural world.^35^ The dualist must contend with explaining such mental states as something that some have, and others do not, in contexts that have profound ethical consequences.^36^ The study of neuroethics, however, is conspicuously absent from the one health movement.^37^

Moral status may be defined as categorical, sentient, or rational. A categorical approach (in the sense that “human moral status” is a prescriptive norm) is often used to distinguish the moral worth of human beings from (whatever other value is common to) all other species.^38^ Culture, therefore, is “what people are” as “constructed by people,”^39^ so that an ethical society may accord absolute value to its own kind (i.e., on the basis that we cannot deny *any* human being full personhood), and thereby justify institutions that unconditionally protect and promote these rights.^40^ Because environmental dualism materializes “at the human–animal interface,” public health suggests that individual agency is (or definitive mental states are) less relevant to achieve categorical targets for health (i.e., human communities): it is meant to be inclusive of all or most human beings.^41^ Personhood, in this regard (in normative democracies), is often specified by the outcomes of deliberative ethics; and society’s “laws” emerge as positivist toward “legal persons” who are defined by convention, tradition, or sentiment. Thus, by evoking the partial idea that nature is a “social construct based on human expectations and knowledge,”^42^ the discursive grounding of ethical frameworks helps implement governance of environmentally friendly policies.^43^ In terms of nature’s relative value to humanity, we are not meant to have naturally *egalitarian* obligations toward it.

Neuroethics might usefully reframe the one health debate by challenging the assumptions of environmental dualism, however: “deepening understanding of the ways that nervous systems and brains are involved in (or evoke) those characteristics that are valued in individuals, groups, and a species should compel and sustain the ways that the organisms that possess such characteristics are regarded and treated.”^44^ Neuroscience, in this respect, justifies using an interspecific concept such as agency—a concept “less speciesist and less blind to the moral importance of abstract or dispersed aspects of nature such as ecosystems, habitats, species or biodiversity.”^45^

Agency refers to the morally relevant attributes of an entity, that may be, but are not necessarily generalizable, to a group or species.^46^ Unlike categorical “persons,” agents are defined by objective status attributes. So although the agency is a biological-related-to-psychological phenomenon (thus is indicative of sentience), its uniquely practical logocentric meaning (that it is rationality-centered) distinguishes “persons” from “things.”^47^ Rational nature signifies an ability to employ means in pursuit of ends freely chosen.^48^ So, assuming agents have the capacity for practical rationality, their actions are ethical or reasonable when they recognize all agents as equal in dignity and rights^49^: agents morally take “due account of the interests of other persons, respecting their right as well as one’s own and maintaining a certain equitableness or mutuality of consideration between oneself and others.”^50^ As such, the “final worth” of the individual is the necessary reason for addressing them as *moral* beings, in ways that are egalitarian toward their *natural* rather than just *human* rights.^51^ We must therefore use impartial description of equivalence with respect to generic goods, and in this respect, although all agents have equal rights, they have different needs with respect to capabilities (and access to goods); such discernment is the subject of theories of justice.^52^ This dialectic approach is dualistic, but only in the nonhumanistic way just specified; and, now makes justice central to the one health narrative.^53^

Agency has two implications for one health. First, any salient specialized adaptations (e.g., grammatical language, capacity to detect cheaters in social contracts, creativity, specialized tool inventions and use) would presuppose capacities for agency, rather than mere species membership.^54^ Neuroscience, in these respects, is beginning to define the (fuzzy) boundaries of interspecies capabilities, including the existence of other (nonhuman) cultures, and certain aspects of learning, communication, and problem-solving in animals that are indicative of agency.^55^ But in neuroscience, in many cases, it is uncertain whether agency exists outside of human experience. And neuroethics has not determined interspecific interests either, yet. It is likely that whatever we discover in these respects, there will be nuances when comparing capacities between species.^56^ A neuroethics approach to one health ethics would therefore suggest that functional neuroanatomy and behavioral analogies contribute to our understanding of agential capabilities and needs, in terms of defining objective health interests, and therefore the rights of and between species.^57^

Second, mental states are inaccessible to physicalist-based study. So, inference and analogy imply that the comparative mental states might be generalizable to (some) other species, and these deductions require logical semblance through interconnectedness and interdependence (knowledge of evolution, biology, and ecology). Therefore, mind, self-awareness, or consciousness are ostensibly underpinning generic components of health; for example, mental illness, illness-induced unconsciousness, or an inclination to be healthy. So, insofar as one health in practice concerns the interests of different species connected to and affecting each other, agency would seem to be relevant to the objectivity of health.^58^ Therefore, a neuroethics of one health presumes that “people” (*qua* human beings) and “animals” (*qua* [nonhuman] animals) have comparable health states. These health states can be described as connected or shared phenomena, which are suggestive of population or public health.^59^ But, one health ethics, rather than an approach to the health determinants between human publics, is coextensively determinate of interspecific well-being.

A neuroethical approach to one health could target issues like pollution, which are disruptive, pervasive, and lasting events that undermine *natural* rights by manifesting as interspecific health determinants: Someone is responsible for its direct health effects, as well as its impact vitiating homes, communities, and environments. In response to these cultural transgressions and other naturally occurring disasters, an egalitarian approach to environmental health justifies, for example, creating a shared biobanking resource so that clinical and veterinarian data are collected and stored, so they can be used to co-investigate and mutually respond to such health impacts,^60^ or support clinical responses such as vaccinating nonhuman animals in equal regard, so that health is improved for all agents in the affected space.^61^

Although I consider a rights approach has merit, ultimately, as a grounds for progressive egalitarianism, it requires adherence to the ethical conditions for ethical agent–agent interactions (and the inferred obligations of ethical institutions),^62^ irrespective of the *isolated* consequences for human interests: *Agency requires that practical judgments are matters of freedom and well-being, so that animal health is potentially and practically an equal moral consideration.* Interspecific rights are controversial because isogenous consistency would require, for example, changing the purpose of conservation policy and practice,^63^ and potentially far-ranging shifts in our treatment of animals,^64^ and (if agency is a valid critique of that treatment) would preclude many aspects of public health that we currently depend upon.^65^ So, conflicts of rights might undermine cultural institutions that protect human rights.^66^ Moreover, that would likely garner resistance in some sectors such as agriculture, husbandry, and medical research, thus undermining the progress made in practical one health to date.

Increasingly, however, such resistance may be seen as illogical and illegitimate.^67^ If agency necessarily grounds mutual recognition of rights, then it also necessarily implicates forms of cooperation.^68^ In ultrasocial cultures, these relationships are codified as norms or theories of justice, but prosociality is also found in nature.^69^ So, if sociality (rather than an agency) is considered *necessary and sufficient* for moral status, then relying on the significance of social scalability should not also commit us to species chauvinism.^70^ In this respect, it is becoming less prudent to exclude at least some nonhuman species as rights holders (if you like, as “social,” rather than *ultra*social animals), and, if interspecific agency is plausible it would narrow, if not collapse, the perceived moral gap between culture and nature. Arguably, as a result, one health and public health would then be part of the same justice discourse about the determinants of health.

## One Health Ethics and Reasonable Environmentalism

Agency also underscores a practicable refocus on the *reasonable* connections made *by* human beings to nature^71^: “Some form of anthropocentrism is a necessary presupposition of any moral theory or moral discourse: no agents, no morality.”^72^ That is, to deny that we are rational is to deny that we are human; and, one might suppose, human beings require nature to flourish. Suppose, then, if there is no nature, there is no agency. This claim assumes that agency is evolved,^73^ but although culture originates in biology (i.e., as a consequence of, and therefore found in nature, as the previous argument suggests), *and* it is passed on between generations and projected into the future, the focus shifts to how culture develops from knowledge, and is practiced ethically through trending values.^74^ Specifically, “climate events and associated suffering can no longer be cast as acts of God or nature. They are now at least partly linked to human agency and responsibility.”^75^ Such impacts on the environment are “the consequence, intended or otherwise, of decisions taken by human minds”^76^; and measuring them is suggestive of the Anthropocene: that human action will be recorded in the geology of Planet Earth.^77^

The “agential” perspective reminds us that “rational natures are not only agents but are on the receiving end of one another’s action. This presupposition is not self-evident: it holds only if rational natures lead connected lives, or (as Kant often puts it) ‘share a world.’”^78^ In this respect, public health refers to *all* human beings by embedding “legal persons” in social and protected contexts in which they live (and the beings they live with).^79^ That view is challenged by versions of environmental naturism, which considers nature to have an inherent value (to various degrees) and therefore that value should be material to what we do.^80^ This conflict becomes obvious, for example, when public health *necessarily* excludes nonhuman species from practical considerations (such as culling policies).^81^ In these contexts, public health’s only referent is the science of animal welfare, and is not meant to carry theoretical animal rights. In law, it is often the perceived connection a nonhuman species or individual animal has to humanity, or a particular human being, that creates responsibility, affords culpability, and provides a remedy.^82^ Law in this sense is not *for* animals, but protects *our* special interests in them; likewise, environmental protection (mainly) benefits humans. Public health also defines public engagement as a method to articulate a *political* conception that accommodates a plurality of comprehensive doctrines, but it is not meant to speak to animal interests because it is not obvious that our interests “overlap.”^83^ As such, a perceived irresolvable conflict between human health and animal welfare plausibly explains a traditional *humanistic* connection to nature.

One health ethics, however, is the emergent idea of reframing positive environmental change as the *principle of natural connection*: It necessarily connects culture to nature in the only world we know to exist.^84^ The world is our (only) life support, *and we know that.* Moreover, since this fact is known, (morally) we have duties to modify our behavior to minimize or mitigate the negative impacts we have on an environment that *we know other agents (or communities) inhabit.* ^85^ Human agents, therefore, must look ahead to the things that are necessary for their and rationally, others’ well-being,^86^ and recognize the impacts in their own actions and beliefs of causing or contributing to biodiversity loss, overconsumption of ecoservices, and capture of public goods.^87^ It is in this respect that individuals have to reevaluate their relationship to nature, perhaps by first imagining a world without connections to green spaces, trees, grass, wilderness, and the creatures found there.^88^ It is in this respect that one health ethics evokes the public interests, in the sense that our actions toward nature may be construed as (un)ethical (or [un]lawful) acts as they appeal to natural rights.^89^ The scope of the public interest (as it is presently understood as a factor pertaining to human rights), however, suggests that the public good—and public bads—can only be generated by human activity: So, cultural artifacts are opportunities to support human well-being, and “captured goods” are such that they decrease our freedom.^90^ One health ethics, for want of a better term, shifts considerations of the public interest to “the natural interest.” This theoretical contention is likely to puzzle one health practitioners and advocates in practice, but I wonder how else they can define a public interest that equally regards animal and environmental interests?

The indirect approach to environmental responsibility, rather than the direct application of rights, will always be subjective: yet, if “…the concept of ‘nature’ is more complex and abstract than it seems, the ecological crisis remains a concrete and empirical reality, now affecting everybody whatever be their vision of nature.”^91^ Suffice to say that, although there is no one concept of ethical environmentalism acceptable to all (yet), perhaps inharmoniousness is itself of value if it has the practical consequence of trying out evermore environmentally friendly policies (i.e., groups achieve similar endpoints, but in different “theoretical” ways). But we should not rest easy on this pragmatic implication, if it is just as likely that the plurality of normative conceptions are themselves in relative conflict^92^; we will still be left with such problems that affect *all* of humanity and the natural world, and a hodgepodge of possible solutions with relative, temporal, and contextual advantages and disadvantages.^93^ These disagreements will then return to questions about how animals and the environment factor in “our” political and social solutions. But, as I have suggested, a philosophical approach to agency may get us closer to a coherent concept of one health. This approach allows the use of common philosophical tools such as “the principle of noncontradiction,” “consistency,” and “ought implies can” (“if you [ethically] should do X, then you [practically] can do X”^94^: a practical rule that you cannot expect people to do something if it is not possible for them to do it, which, I think, speaks to the pitfalls of public health, too). These logical rules may have an impact on policy if embedded in ethical norms, thus making the case for environmentalism plausible, convincing, attractive, and possible, on grounds already established as reasonable. Much more work is needed in this regard, principally in the ways that the public interest defines the social value (the public good) of environmentalism, the specific function of cooperation between species, and the human species’ specific role in modern nature.^95^

Finally, what I hope I have established is that agency creates a logical space for one health ethics coextensive to public health, so that both public health and one health ethics are compatible in the discourse about social regulation.^96^ One health, therefore, becomes a lens—a perspective, personal narrative, and field of study—to identify actions that are hostile toward interspecies well-being, and reason to commit to better ways of living with nature.^97^

## Conclusion

Environmental dualism has been used in this article to differentiate a humanistic understanding of “cultural” rights, as specific to public health, from the naturalistic rights in one health. I have not attempted to settle the matter of dualism, but the dichotomy is illustrative of the fact that, in public health, nature has no value independent of providing healthy and sustainable ecoservices. Strong dualists claim that such services are meant to be used and potentially used up if replacements and alternatives are available. That can lead to exploitation and capture, regardless of temporal and contextual environmentally friendly outlooks bridging cultural and natural realms. These consequences have been underexplored in the ethics of one health.

My goal in this article is a call for recognition of the unique, essential, and necessary relationship agents have with nature, and, as such, to present an opportunity to foster better ways of living with and within it. One health is coextensive to public health, so it substantiates environmental responsibility as a public good, and frames public bads as collectively irresponsible or exploitative. In this respect, one health has two specific connotations. First, one health is a way to narrate the ethical conditions necessary to support natural phenomena as the public good. That suggests that there is necessarily interpersonal comparability between agents—and therefore potential interspecies comparability. The neuroscience of one health ethics, therefore, is grounded in an equal and equivalent ethical connection between the planet’s life support for all life and the environment’s cultural value to us. Second, one health is a theory of environmentalism: It is part of the neuroethical study of rational choice, and about discovering opportunities to live as a natural egalitarianist, to be expressive of the value of nature, and act mutually beneficially toward others living in shared natural spaces. Our gaze toward nature should therefore necessitate continuous, context-specific investigation to discover ways of (and barriers to) living, without costs and compromises, that forsake nature for purely humanistic imperatives. If outcomes are meant to be anthropocentric, in this regard, then they are properly described as public health.

## Notes

1. Public health is the organized effort of society to keep people healthy and prevent injury, illness, and premature death. One health is used in this respect as a framework to enable mechanisms of coordination and partnership to build more collaborative public health systems. “One health in all policies” is meant to address inequality and inequities in “…all social, economic, and environmental health determinants, and to promote sustainable well-being for the population.” It does, however, suggest an “an integrated understanding of health that is not limited by species boundaries and seeks to bring together sectors to better address the health impacts of wide-scale environmental change resulting from human activity.” The World Bank. *One Health Operational Framework for Strengthening Human, Animal and Environmental Public Health Systems at their Interface.* Washington, DC: World Bank Group; 2018, at 4, 102.

2. For background, see Coghlan S, Coghlan B. One health, bioethics, and nonhuman ethics. *The American Journal of Bioethics* 2018;**18**:3–5.

3.

Verweij M , Bovenkerk B. Ethical promises and pitfalls of onehealth. Public Health Ethics 2016;9:1–4 . Also see, “After all, practitioners rather than philosophers may be better placed to understand the political and strategic climate in which public health decision making operates”; Johnson J , Degeling C. Does one health require a novel ethical framework? Journal of Medical Ethics 2019;45:239–433077284110.1136/medethics-2018-105043.

4.

Capps B , Bailey M , Bickford D , Coker R , Lederman Z , Lover A , et al. Introducing one health to the ethical debate about zoonotic diseases in South East Asia. Bioethics 2015;29:588–962567589910.1111/bioe.12145PMC7161875.

5.

Beever J , Morar N. The epistemic and ethical onus of ‘one health’. Bioethics 2019;33:185–943034191610.1111/bioe.12522.

6. Environmental dualism suggests that “…we do not live in nature”; Katz E. Geoengineering, restoration, and the construction of nature: Oobleck and the meaning of solar radiation management. *Environmental Ethics* 2015;**37**:485–98, at 489.

7.

Lysaght T , Capps B , Baily M , Bickford D , Coker R , Lederman Z , et al. Justice is the missing link in one health: Results of a mixed methods study in an urban city state. PLoS One 2017;12(1):e017096710.1371/journal.pone.0170967PMC527136128129409.

8.

Craig S. Toward a modernized definition of wildlife health. Journal of Wildlife Diseases 2014;50:427–302480717910.7589/2013-11-305.

9.

Giordano J , Becker K , Shook, J. On the “neuroscience of ethics” - Approaching the neuroethical literature as a rational discourse on putative neural processes of moral cognition and behavior. Journal of Neurology and Neuromedicine 2016;1:32–6.

10.

Boylan M. ‘On pain of contradiction’ A key moment in deductive agency arguments. In Bauhn P , ed. Gewirthian Perspectives on Human Rights. Oxon: Routledge; 2016:46–62 , at 47.

11. Ducarme F, Couvet D. What does ‘nature’ mean? *Palgrave Communications* 2020;614. doi:10.1057/s41599-020-0390-y.

12. Culture involves ideas, practices, skills, attitudes, norms, art styles, technology, ways of speaking, and other “symbols and values” that change through time, but persist in knowledge and traditions; [based on the arguments of] Dennett D. *Darwin’s Dangerous Idea: Evolution and the Meanings of Life.* London: Allen Lane; 1995.

13.

Richerson P , Boyd R , Henrich J. Gene-culture coevolution in the age of genomics. Proceedings of the National Academy of Sciences USA 2010;107(Suppl):8985–9210.1073/pnas.0914631107PMC302402520445092.

14. Heath J, Rioux C. Recent trends in evolutionary ethics: Greenbeards! *Biology & Philosophy* 2018;33:16. doi:10.1007/s10539-018-9627-1.

15. For a scientific account, see “The ability to establish the connection between means and ends happens to be the fundamental intellectual capacity that has made possible the development of human culture and technology”; Ayala F. The difference of being human: Morality. *Proceedings of the National Academy of Sciences USA* 2010;107:9015–22, at 9018. For a philosophical account, see Haksar V. *Equality, Liberty and Perfectionism.* Oxford: Clarendon Press; 1979.

16. “…the vision of human beings as a distinct order of creation, the guardians of the natural order, answerable for their lives and duty bound to make the best of them”; Scruton R. Animal rights. *City Journal* 2000;Summer; available at https://www.city-journal.org/html/animal-rights-11955.html (last accessed 21 Jan 2022).

17.

Stucki S. Towards a theory of legal animal rights: Simple and fundamental rights. Oxford Journal of Legal Studies 2020;40:533–603331206810.1093/ojls/gqaa007PMC7714088.

18.

Compare Stone C. Should Trees Have Standing? Law Morality and the Environment. 3rd ed. Oxford: Oxford University Press; 2010 .

19.

Marmot M , Wilkinson R. Social Determinants of Health. Oxford: Oxford University Press; 2006 .

20. See Hailwood S. Alienation and natures. Environmental Politics 2012;21:882–900 .

21. Lee L. A bridge back to the future: Public health ethics, bioethics, and environmental ethics. *American Journal of Bioethics* 2017;**17**:5–12. See also Potter V. Fragmented ethics and ‘bridge bioethics’. *Hastings Center Report* 1999;**29**:38–40. Dualism may also be construed as the perceived scholarly divide between science and humanities, that has been critiqued under unifying environmental narrative: how we currently generate and use data creates disciplinary silos, leading to superficial understanding that impedes awareness of nature’s importance to our wellbeing; Leach M, Scoones I. The social and political lives of zoonotic disease models: Narratives, science and policy. *Social Science and Medicine* 2013;**88**:10–17. Also see note 4, Capps et al. 2015.

22. The public good is a collective rights claim to distributive goods, which serves to increase equality of welfare opportunities; the public interest establishes whether the current or imagined provision of such goods is ethical (or legal) in respect to rights; see Capps B. Public goods in the ethical reconsideration of research innovation. In: Capps P, Pattinson S, eds. *Ethical Rationalism and the Law.* Oxford: Hart; 2016:149–69.

23.

Degeling C , Johnson J , Kerridge I , Wilson A , Ward M , Stewart C , et al. Implementing a one health approach to emerging infectious disease: Reflections on the socio-political, ethical and legal dimensions. BMC Public Health 2015;15:1307 2671506610.1186/s12889-015-2617-1PMC4696140.

24. “Incorporating the perspective of sustainability into our cultural paradigm will support continued improvement in human health and well-being, as well as environmental resource protection and economic prosperity, now and in the future”; Munns W, Rea A, Mazzotta M, Wainger LA, Saterson K. Toward a standard lexicon for ecosystem services. *Integrated Environmental Assessment and Management* 2015;**11**:666–73, at 666.

25. Cultural environmentalism refers to an “ecology” of cultural production and creativity, that is, rooting sociality in the quality of the human environment, and protecting (“conservation” of) the public domain; see Boyle J. The second enclosure movement and the construction of the public domain. *Law and Contemporary Problems* 2003;66:33–74. My use of the term is also based on the traditional sense of environmentalism: a “political and ethical movement that seeks to improve and protect the quality of the natural environment through changes to environmentally harmful human activities; through the adoption of forms of political, economic, and social organization that are thought to be necessary for, or at least conducive to, the benign treatment of the environment by humans; and through a reassessment of humanity’s relationship with nature.” Elliott L. Environmentalism. *Encyclopaedia Britannica*; available at https://www.britannica.com/topic/environmentalism (last accessed 21 Jan 2022).

26. See Degeling C , Dawson A , Gilbert G. The ethics of one health. In: Walton M , ed. One Planet, One Health. Sydney: Sydney University Press; 2019:65–84.

27. Wallace G, Bergmann L, Kock R, Gilbert M, Hogerwerf L, Wallace R, et al. The dawn of structural one health: A new science tracking disease emergence along circuits of capital. *Social Science & Medicine* 2015;**129**:68–77.

28. See Rock M. Who or what is ‘the public’ in critical public health? Reflections on posthumanism and anthropological engagements with one health. Critical Public Health 2017;27:314–24.

29. “Climate change, biodiversity loss and degradation of ecosystems are interdependent and pose significant societal challenges, threatening economic and social stability, public health and [human] well-being”; European Environment Agency. *Nature-Based Solutions in Europe: Policy, Knowledge and Practice for Climate Change Adaptation and Disaster Risk Reduction.* EEA Report No 01/2021. Luxembourg: Publications Office of the European Union; 2021, at 11.

30.

Callicott J. Thinking Like a Planet: The Land Ethic and the Earth Ethic. Oxford: Oxford University Press; 2013 , at 54.

31.

Donaldson S , Kymlicka W. Zoopolis: A Political Theory of Animal Rights. Oxford: Oxford University Press; 2011 .

32. Culture is also a simplification that allows a few to *dominate and exploit* nature so that *they* can prosper; Midgley M. Selfish genes and social darwinism. *Philosophy* 1983;**58**:365–77. See note 18, Stone 2010.

33. For example, the rational nature of human beings and dolphins (*Tursiops truncates*) are “consciously” similar, but (for example) have quite different physiological needs. So that raises questions about how comparability between species accounts for their moral status and different experiences of “health”; see Herman L. Intelligence and rational behaviour in the bottlenosed dolphin. In: Hurley S, Nudds M, eds. *Rational Animals?* Oxford: Oxford University Press; 2006:439–67. Such deductions are not purely scientific observations, because, for instance, between and within species, categorical groupings often break down due to marginal, potential, proportional and future considerations. That is, the difference between temporal functional states—between human embryos and born human beings, inferred cognitive states of individuals, for example, sleeping or unconsciousness—or of the unusual, for example, Kanzi the bonobo (*Pan paniscus*), using human language; for background, see Lafollette H, Shanks N. The origin of speciesism. *Philosophy* 1996;**71**:41–61.

34. “There are of course sub-categories of meaning that are markedly anthropocentric. The terms ‘industrocentrism,’ ‘human chauvinism,’ and ‘speciesism’ are key examples”; Kopnina H , Washington H , Taylor B , Piccolo JJ . Anthropocentrism: More than just a misunderstood problem. Journal of Agricultural and Environmental Ethics 2018;31:109–27.

35.

Dobzhansky T. The Biology of Ultimate Concern. New York: New American Library; 1967 .

36. For a discussion about how culture and nature are understood through theories of mind, see: Crooks M. Consciousness: Sentient and rational. The Journal of Mind and Behavior 2011;32:251–75.

37.

Altman M. Decentering anthropocentrisms: A functional approach to animal minds. Between the Species 2015;18:27–50 .

38. See “Ye are of more value than many sparrows”; Donagan A. *The Theory of Morality.* Chicago: University of Chicago Press; 1977, at 171.

39. A “sustainable public requires the ‘shared imagination’ of its constituents; a shared belief in the fact of, and in the broad terms the nature of, their association”; Coggon J. *What Makes Health Public?* Cambridge: Cambridge University Press; 2012:212–4.

40. In the sense that species membership overrides other moral demands and has force independent of objective factors. John Coggon claims that “the population the [public health] approach speaks to includes all born, living human beings within the jurisdiction of a Liberal State… and denies *special* normative protection to potential members that some theorist would include” (citing Peter Singer’s *Animal Liberation*); See note 39, Coggon 2012, at 212–13. For a philosophical approach to this point, see note 15, Haksar 1979, at 66; and see note 38, Donagan 1977. A categorical approach could also create a single “land”, life, or bios—depending on how one “speaks”; see Leopold A. “The Land Ethic,” A Sand County Almanac: And Sketches Here and There. London: Oxford University Press; 1949:201–26; Zagorac I. Fritz Jahr’s bioethics imperative. Synthesis Philosophica 2011;51:141–50.

41. See note 1, The World Bank 2018, at 4.

42. Although health can be a biological phenomenon regardless of the species, the *humane response* to prevention and treatment is a cultural responsibility; see note 8, Craig 2014.

43.

Brulle R , Jenkins J. Spinning our way to sustainability? Organization and Environment 2006;19:82–7, at 84. Also see “solutions that work and are acceptable for all involved”; Loman O. A problem for environmental pragmatism: Value pluralism and the sustainability principle. *Contemporary Pragmatism* 2020;**17**:286–310, at 287.

44.

Loveless S , Giordano J. Neuroethics, painience, and neurocentric criteria for the moral treatment of animals. Cambridge Quarterly of Healthcare Ethics 2014;23:163–722449571310.1017/S0963180113000698, at 164.

45.

O’Neill O. Kant on duties regarding nonrational nature. Aristotelian Society Supplementary *Volume* 1998;72:211–28, at 211. Also see Beyleveld D. Williams’ false dilemma: How to give categorically binding impartial reasons to real agents. Journal of Moral Philosophy 2013;10:204–26.

46.

Capps B. Do chimeras have minds? The ethics of clinical research on a human-animal brain model. Cambridge Quarterly of Healthcare Ethics 2017;26:577–912893734210.1017/S0963180117000093.

47. See note 45, O’Neill 1998, at 214.

48. See note 45, Beyleveld 2013.

49.

Beyleveld D. The principle of generic consistency as the supreme principle of human rights. Human Rights Review 2012;13:1–18 .

50.

Gewirth A. The rationality of reasonableness. Synthese 1983;57:225–47, at 225.

51.

Toddington S. Agency, authority, and the logic of mutual recognition. Ratio Juris 2013;28:89–109 .

52. See de Maagt S. Korsgaard’s other argument for interpersonal morality: The argument from the sufficiency of agency. Ethical Theory and Moral Practice 2018;21:887–902 .

53. See note 7, Lysaght et al. 2017.

54. See note 49, Beyleveld 2012.

55.

Buller T , Shriver A , Farah M. Broadening the focus. Cambridge Quarterly of Healthcare Ethics 2014;23:124–82449561810.1017/S0963180113000650.

56.

Pattinson S , Beyleveld D. Defending moral precaution as a solution to the problems of other minds: A reply to Holm and Coggon. Ratio Juris 2010;23:258–73.

57. See note 9, Giordano et al. 2016.

58. That all ostensible agents must be treated as equal in rights (subject to the principle of equality), and equivalently in respect to the claims to the benefits of rights (the principle of equivalence), disproves the premise that environmentalism must be approached as a matter solely effecting human beings; see Capps B. One health ethics. *Bioethics* 2022;**36**:348–55. doi:10.1111/bioe.12984.

59.

Rabinowitz P , Odofin L , Dein J. From “Us vs. them” to “Shared risk”: Can animals help link environmental factors to human health? EcoHealth 2008;5:224–91878792410.1007/s10393-008-0170-4.

60.

Capps B , Lederman Z. One health and paradigms of public biobanking. Journal of Medical Ethics 2015;41:258–62.2457039510.1136/medethics-2013-101828

61.

Capps B. Lederman Z . One health, vaccines and Ebola: The opportunities for shared benefits. Journal of Agriculture and Environmental Ethics 2015;28:1011–3210.1007/s10806-015-9574-7PMC708832532214867.

62.

Gewirth A. Reason and Morality. Chicago: Chicago University Press; 1978 .

63. Whiten A. The burgeoning reach of animal culture. *Science* 2021;372(6537):eabe6514. doi:10.1126/science.abe6514.

64. See note 17, Stucki 2020.

65. “Recognising that our views … might be shaped by internal biases, including a possible anthropocentric approach that places more value on humans than animals, ecological systems, and biodiversity…”; Khan M , Rotherman-Ostrow P , Spencer J , Hasan N , Sabirovic M , Rahman-Shepherd A , et al. The growth and strategic functioning of one health networks: A systematic analysis. Lancet Planet Health 2018;2:e264–73 2988015810.1016/S2542-5196(18)30084-6.

66.

Campbell S , Hoey A , Maynard J , Kartawijaya T , Cinner J , Graham NAJ , et al. Weak compliance undermines the success of no-take zones in a large government-controlled marine protected area. PLoS One 2012;7(11):e50074 2322623710.1371/journal.pone.0050074PMC3511441. Such resistance is an ethical concern in all deliberative endeavors; see Capps B. Gene drive gone wild: Exploring deliberative possibilities by developing one health ethics. Law, Innovation and Technology 2019;11:231–6.

67. Medina M. Law change sees pets in Spain considered sentient beings, with welfare taken into account should a couple separate. *El Paīs* (Madrid) 2022 Jan 5. In this respect, “cultural” environmentalism might ethically (coextensively) exclude considerations of natural justice; Klinsky S. Why equity is fundamental in climate change policy research. Global Environmental Change 2017;44:170–3.

68. Also see Health J. The benefits of cooperation. Philosophy and Public Affairs 2006;34:313–51.

69.

Segovia-Cuéllar A , Del Savio L. On the use of evolutionary mismatch theories in debating human prosociality. Medicine, Health Care and Philosophy 2021;24:305–143398219110.1007/s11019-021-10025-4PMC8349330.

70. See Routley R , Routley V. Against the inevitability of human chauvinism. In: Goodpaster K , Sayre K eds. Ethics and Problems of the 21st Century. Notre Dame, IN: University of Notre Dame Press; 1979:36–59 .

71. See note 50, Gewirth 1983.

72. See note 45, O’Neill 1998, at 217.

73. See note 14, Heath, Rious 2018.

74. See generally Beyleveld D , Düwell M , Spahn A. Why and how should we represent future generations in policymaking? Jurisprudence 2015;6:549–66.

75.

Ribot J. Cause and response: Vulnerability and climate in the anthropocene. Journal of Peasant Studies 2014;41:667–705 , at 667.

76. Hamilton C. Human destiny in the anthropocene: A speech to the conference ‘Thinking the anthropocene’. *Sciences Po*, Paris, 15 November 2013; 2014; available at http://www.institutmomentum.org/wp-content/uploads/2014/04/Human-Destiny-in-the-Anthropocene.pdf (last accessed 21 Jan 2022).

77. These consequences include the passive role of nature in achieving *our* ends (i.e., farmed animals and methane production, or intruding in wild places that harbor zoonotic risks); see Keulartz J, Bovenkerk B. Animals in our midst: An introduction. In: Bovenkerk B, Keulartz J, eds. *Animals in Our Midst: The Challenges of Co-existing with Animals in the Anthropocene.* The International Library of Environmental, Agricultural and Food Ethics. Vol. 33. Cham: Springer; 2021.

78. See note 45, O’Neill 1998, at 218.

79. See especially: “What is needed is a clear vision of, and a rationale for, healthy populations as a political priority”; Gostin L, Wiley L. *Public Health Law: Power, Duty, Restraint.* Oakland, CA: University of California Press; 2016, at 7.

80.

Gewirth A. Human rights and future generations. In: Boylan M , ed. Environmental Ethics. 2nd ed. Oxford: Wiley Blackwell; 2014:118–22.

81.

Lederman Z , Magalhães-Sant’Ana M , Voo TC . Stamping out animal culling: From anthropocentrism to one health ethics. Journal of Agricultural and Environmental Ethics 2021;34:27 3448369610.1007/s10806-021-09868-xPMC8403469. doi:10.1007/s10806-021-09868-x.

82. See note 18, Stone 2010.

83. This suggests a distinction between philosophical (ethnos) and political (public—demos) versions of “reasonableness”; see generally Claassen R , Düwell M. The foundations of capability theory: Comparing Nussbaum and Gewirth. Ethical Theory and Moral Practice 2013;16:493–50.

84. See note 58, Capps 2022.

85. See note 45, Beyleveld 2013.

86. “…the validity of such relations in community is the essential political expression of that [agential] perspective”; See note 51, Toddington 2013, at 108.

87. See Brudner A. Constitutional Goods. Oxford: Oxford University Press; 2004 .

88. See Zwarthoed D. Should future generations be content with plastic trees and singing electronic birds? *Journal of Agriculture and Environmental Ethics* 2016;29:219–36. Agency therefore signifies a basic responsibility for one’s actions, but therein resides (beyond this article) the complex relationship between psychology, sociology, and the state, such that an ethical agent may ask nonetheless “I can’t do that”, as well as “What good would it do, anyway?” (referring to the plausible things one can do); see generally, Kemp P , Nielsen L. The Barriers to Climate Awareness. Copenhagen: Ministry of Climate and Energy; 2009.

89. See note 22, Capps 2016.

90. See note 22, Capps 2016.

91. See note 11, Ducarme, Couvet 2020, at 5.

92. See note 66, Capps 2019.

93.

Gewirth A. Is cultural pluralism relevant to moral knowledge? Social Philosophy and Policy 1994;11:22–43 .

94.

Gewirth A. The ‘Is-Ought’ problem resolved. Proceedings and Addresses of the American Philosophical Association 1973;47:34–61 .

95. The physical undertaking of that work might be a practical justification for the novelty of one health, but as such, is open to misuse if there is no normative criterion to harmonize everyone’s ethical claims; see generally Spencer J, McRobie E, Dar O, Rahman-Shepherd A, Hasan N, Hanefeld J, et al. Is the current surge in political and financial attention to one health solidifying or splintering the movement? *BMJ Global Health* 2019;4:e001102.

96.

Brownsword R. Public health interventions: Liberal limits and stewardship responsibilities. Public Health Ethics 2013;6:235–40.

97. In this respect, *universal goods* identify the preconditions for *all* life. These objective, positive goods are relevant for all species, and, as such contrast with “public” or universal bads such as habitat loss. For example, humans require portable water, and Bottlenose dolphins (see note 33) require unpolluted seas, so that water—in this sense, as a basic good—benefits them both. However, trees also benefit from unpolluted ground water, thus suggesting the idea of water as a universal good. See note 61, Capps, Lederman 2015.

